# Rehabilitation of facial nerve palsy combining neuromuscular retraining and botulinum toxin A injection: a tertiary referral centre experience and a new working protocol proposal

**DOI:** 10.1007/s00405-025-09465-y

**Published:** 2025-05-22

**Authors:** Marco Bonali, Federico Calvaruso, Andrea Tozzi, Cinzia Del Giovane, Federica Nizzoli, Elena Reggiani, Alfredo Lo Manto, Martina Silvestri, Daniele Marchioni, Giuseppe Ferrulli, Claudio Melchiorri

**Affiliations:** 1 Department of Otorhinolaryngology - Head and Neck Surgery, University Hospital of Modena, 41124 Modena, Italy; 2 Department of Otorhinolaryngology - Head and Neck Surgery, Ospedale Infermi, 47900 Rimini, Italy; 3 Department of Medical and Surgical Sciences for Children and Adults, University Hospital of Modena, 41124 Modena, Italy; 4 Institute of Primary Health Care (BIHAM), University of Bern, 3012 Bern, Switzerland; 5 Otolaryngology Unit, Department of Surgery, Azienda USL-IRCCS di Reggio Emilia, 42123 Reggio Emilia, Italy

**Keywords:** Facial nerve palsy, Synkinesis, Neuromuscular retraining, Botulin toxin type A

## Abstract

**Purpose:**

Neuromuscular retraining (NMR) is the primary treatment for synkinesis in facial nerve palsy (FNP) patients, with botulinum toxin type A (BTX-A) enhancing rehabilitation outcomes. This study aims to assess the functional outcomes of combining BTX-A infiltration and NMR in treating synkinesis in peripheral FNP patients.

**Materials and methods:**

This retrospective study, conducted at the University Hospital of Modena (2020–2023), included patients with chronic peripheral FNP treated with NMR and at least two BTX-A injections for synkinesis. The degree of paralysis was assessed using the Sunnybrook classification system before and after BTX-A injections.

**Results:**

The study included 140 patients, with FNP causes categorized as iatrogenic (46%), idiopathic (38%), infective (10%), and post-traumatic (5.3%). The median time between paralysis onset and first BTX-A injection was 19.3 months, with an average of 15.68 IU per injection session. The most commonly treated muscles for synkinesis were the platysma, mentalis, orbicularis oculi, orbicularis oris, and levator labii superioris alaeque nasi on the affected side. For facial symmetry, the frontalis, zygomatic, depressor labii inferioris, and depressor anguli oris muscles on the healthy side were treated. Following two BTX-A injections, the Sunnybrook score improved by a mean of 11.76 points, and the synkinesis score decreased by 4.78 points (both statistically significant, *p* < 0.05).

**Conclusion:**

BTX-A is an effective treatment for reducing synkinesis and improving facial function in chronic FNP. When combined with consistent rehabilitation, it supports the resumption of voluntary movement. NMR proves to be an essential adjunctive therapy in enhancing the outcomes of BTX-A treatment.

## Introduction

Facial animation plays an indispensable role in human communication, serving as a primary conduit for emotional expression and nonverbal communication. Facial Nerve Palsy (FNP) has been linked to a range of adverse psychological outcomes, including depression, social isolation and a reduced quality of life [1]. The causes of facial paralysis are numerous and heterogeneous. They can be divided into congenital and acquired, the latter being represented by Idiopathic Bell’s Palsy (IBP), Ramsay Hunt Syndrome (RHS) and other infectious (Lyme disease, otitis media), iatrogenic (following otosurgery, lateral skull base and parotid surgery), tumour (malignant parotid lesions, geniculate hemangioma, endolymphatic sac tumour exc.), traumatic (forceps delivery, temporal bone fracture, penetrating wounds, dog bites, stab wounds, and gunshot injuries) [2].

Facial synkinesis represents one of the most distressing consequences of chronic FNP. It is characterised by the combination of an absence of movement on the paretic side and a loss of balance between the paralyzed and moving sides. Synkinesis is defined as an abnormal involuntary facial movement occurring with the voluntary movement of a different facial muscle group [3]. The underlying pathophysiology of facial synkinesis is likely multifactorial, although the predominant mechanism appears to be aberrant regeneration of facial nerve fibres to the facial muscle groups following facial nerve injury [4]. A variety of approaches may be employed in the rehabilitation of synkinesis. Among these, Neuromuscular Retraining (NMR) is of particular importance, facilitating the restoration of intended facial movement patterns and the reduction or elimination of unwanted patterns of facial movement through the redirection of cortical projections to non-synkinetic motoneurons [5].

Botulinum toxin type A (BTX-A) chemical neurectomy has been adopted over the past decade in a variety of clinical settings for the treatment of several conditions, including some forms of blepharospasm, strabismus, and spasmodic dysphonia [6–8]. BTX-A exerts its effects by binding to the presynaptic membrane and inhibiting the release of acetylcholine, thereby paralyzing the skeletal muscles [9]. Despite the absence of codified guidelines, BTX-A has been applied from the early 1990s for the treatment of asymmetries caused by FNP, with promising results and improvements in quality of life [10]. The utilisation of BTX-A for the treatment of post-paralytic synkinesis represents one of the most recent applications in the field of FNP rehabilitation [11]. Furthermore, BTX-A injections may be employed to mitigate the occurrence of facial hypercontractions that may ensue from the spontaneous or rehabilitative restoration of facial movement [12]. The combination of BTX-A and NMR may facilitate a ‘window of opportunity’, during which patients can engage in training with more typical movement patterns, free from the interference of synkinesis, while also experiencing a reduction in synkinesis once drug effects subside [13].

The aim of this study is to assess the impact of combining NMR with a prior BTX-A injection (NMR/BTX-A) on facial synkinesis in individuals with chronic FNP on the Sunnybrook Facial Grading System. Furthermore, this paper aims to present our injection protocol and our preliminary experience with NMR/BTX-A.

## Materials and methods

### Patients

A retrospective review was conducted on 140 patients who underwent combined NMR/BTX-A treatment at the Otolaryngology Department of the University Hospital of Modena between September 2020 and September 2023. The inclusion criteria were as follows: unilateral peripheral chronic FNP without recovery or partially recovered within 12 months since the onset of the palsy; minimum follow-up period of six months since the beginning of NMR/BTX-A treatment; absence of an history of adverse reactions to BTX-A. Additionally, patients who had undergone a previous facial nerve reanimation or static and dynamic techniques of facial nerve rehabilitation were also included. The paediatric population (aged below 18 years), patients with a central origin of FNP and those who were lost to follow-up were excluded from the study.

### Ethical statement

The present retrospective study was approved by the Internal Review Boards (IRB) of the University Hospital of Modena (261/2023/OSS*/AOUMO). The study was performed according to the Declaration of Helsinki. Ethical Committee (ID 6052).

### Assessment of the FNP

All patients were discussed in the multidisciplinary facial nerve office at the University Hospital of Modena. A multidisciplinary team comprising an ENT surgeon, a neurologist and a speech therapist with extensive experience in facial nerve rehabilitation participates in the discussion. The severity of the facial defect was evaluated using the Sunnybrook scale (SBs) [26]. It assesses the symmetry during periods of rest and during voluntary movements, as well as the presence or absence of synkinesis. The Sunnybrook scale (SBs) ranges from 0 to 100. The former value represents a state of complete paralysis, whereas the latter represents a state of normal facial function.

### Intervention (Neuromuscular retraining combined botulin toxin type A Injection)

Following the initial screening, patients deemed suitable for NMR/BTX-A undergo a period of evaluation lasting between three and twelve months. At the preliminary stage the SBs is employed to assess facial movements. Based on the speech therapist’s evaluation, BTX-A injections are administered into the mimic muscles indicated, with the objective of reducing hypertonus, synkinesis and improving symmetry. The treatments are conducted via the administration of Botox^®^ (Onabotulinumtoxin A) at a dilution of 100 U in 4 cc of 0.9% saline solution (concentration 2.5 U/0.1 ml) using a 1 cc syringe with a 30 Gauge needle. The injection points are meticulously delineated in the extant literature, and we have adopted them [16]. It is advised that a period of at least 30 days should occur following the injection during which a complete physical rest is observed, as well as the avoidance of any facial massage and rehabilitative exercise in the following fifteen days. Once this period has ended, a new SBs evaluation is applied, and patients commence the NMR/BTX-A rehabilitation programme. In the initial and second evaluations, patients are instructed in selective motor control techniques by an expert speech-language pathologist, and then encouraged to perform these exercises independently at home. Mirror exercises facilitate the central regulation of motor control based on sensory input, thereby enhancing neural adaptation. The slow contraction allows the patient to make the necessary corrections to the movements, both in terms of velocity and strength. At last, the symmetry of moments permits a physiological activation of the affected side, thereby avoiding muscular over-contraction on the healthy side. Facial massages prevent post-paretic syndrome and mitigate the recruitment and hyperactivity of adjacent muscles, which in turn improves fine mimic coordination.

All the SB’s evaluation has been recorded using a mirrorless digital camera with a 24.2-megapixel resolution (Sony 7 III, Sony, Japan) coupled with a 40 mm F/2,5 Macro 1:1 fixed focal length lens, and are stored on an external memory device protected by a password, which is known only to the personal involved in the study.

### Statistical analysis

Descriptive statistics were employed to characterize the patient cohort. We calculated mean, standard deviation, median and range for continuous variables. Categorical variables were expressed as frequencies and percentages. Parametric paired t-tests were conducted to evaluate whether the rehabilitation outcomes differed before and after NMR/BTX-A, including both SBs and synkinesis. Results were reported as mean difference and relative 95% confidence interval (CI). Statistical analyses were performed using Data analysis was performed using STATA/IC 18 statistical package (StataCorp LP, Texas, USA). We considered a p-value < 0.05 as statistically significant.

## Results

### Study cohort

One hundred and forty patients met the inclusion criteria and were selected for retrospective review of their medical charts. A total of 53 male and 87 female patients (mean age of 55.5 years, standard deviation of 13,5, ranging from 21 to 89 years old) were evaluated by our Facial Nerve Unit between September 2020 and September 2023. Of the cases observed, 69 were on the left side and 71 were on the right. Four patients had a history of prior FNP. In our series of patients, the most common cause of facial nerve palsy was iatrogenic, accounting for 64 cases (46%). Non-iatrogenic causes include idiopathic Bell paralysis (53 cases, 38%), Ramsay Hunt syndrome (15 cases, 10%), and post-traumatic cases (7 cases, 5%). In one patient, the aetiology remained uncertain (1%). In only two cases was BTX-A rehabilitation attempted as an alternative to other rehabilitation strategies. A total of fourteen patients underwent facial nerve reanimation with hypoglossal-facial nerve anastomosis as part of their rehabilitation programme. General features of the population are summarized in Table 1. Of the 140 patients in the initial cohort, only 126 had a complete dataset containing all information related to BTX-A administration. Therefore, these patients constitute the actual study population.

**Table 1:** General features of the study population (*n* = 140)

Characteristics	*n*°	Frequency (%)
Population	140	
Mean age ± SD (range)	55,45 ± 13,5 (21–89)	
Gender	140	
Male		53 (37,86%)
Female		87 (62,14%)
Side	140	
Left		69 (49,29%)
Right		71 (50,71%)
Diagnosis	140	
Cholesteatoma		7 (5,02%)
Lymphangioma		1 (0,71%)
Facial Nerve Haemangioma		2 (1,42%)
Endolymphatic Sac Tumor		1 (0,72%)
Mixed Nerve Neuroma		2 (1,42%)
Adenoid Cystic Carcinoma (Parotid gland)		2 (1,42%)
Recurrent Pleomorphic Adenoma (Parotid gland)		1 (0,71%)
Acoustic Neuroma		48 (34,29%)
IBP		53 (37,86%)
RHS		15 (10,71%)
Traumatic		7 (5,02%)
Unknown		1 (0,71%)
Recurrence of Paralysis		4 (2,88%)
Previous facial reanimation treatment		14 (10%)
Hypoglossofacial Anastomosis		13 (9,28%)
Masseteric- Facial Nerve Anastomosis		1 (0,72%)
Latency to treatment Onset	129	
0–12 months		19 (14,73%)
12–24 months		65 (50,79%)
> 24 months		45 (34,88%)

### BTX-A injections

#### First injection

A total of 126 cases were available for analysis, providing comprehensive data on the initial rehabilitation cycle. The mean total score according to the SBs at the beginning of the treatment was 67.47. The mean total SBs evaluated 30 days following the injection was 76.09, representing an average improvement of 8.62 points (95%CI 7.70 to 9.53, *p* < 0.05). Among the total number of individuals included in the analysis, 66 cases, representing 47% of the population, demonstrated an increase of at least ten points in the SBs. With regard to the mean synkinesis score at the outset of the treatment, this was 9.30, while a score after 30 days of 5.1, showing a mean decrease of 4.17 points (95%CI 3.91 to 4.43, *p* < 0.05).

#### Second injection

A total of 87 cases were identified within the population that had received at least two injections. The mean total SBs prior to the start of treatment was 67.59, while the mean total SBs following the second cycle of rehabilitation was 79.35. The mean improvement was 11.76 (95%CI 10.56 to 12.96, *p* < 0.05). A total of 57 patients (65.52%) demonstrated a minimum improvement of ten points in their score. The mean length of time between two injections was 7.57 months (ranging from 4.54 to 15 months). The mean synkinesis score prior to treatment initiation was 9.42, with an average score after the treatment cycle of 4.64. The mean difference score was 4.78 (95%CI 4.41 to 5.15, *p* < 0.05). The results are reported in Table 2.

**Table 2:** Sunnybrook scale variation along single and double BTX-A injections cycle in total cohort. ΔSB-1 = SB-1post– SB-1pre; ΔSynk-1 = Synk-1post– Synk-1pre. ΔSB-2 = SB-2post– SB-2pre; ΔSynk-2 = Synk-2post– Synk-2pre

Population	Analysis	Mean	Range	SD	CI	*P* value
First Injection (*n* = 126)	SB-1pre	67,47	38–94	12,55	[65,26–69,68]	/
	SB-1post	76,09	49–98	11,48	[74,07–78,11]	/
	ΔSB-1	**8**,**62**		5,19	[9,53 − 7,70]	**< 0**,**05**
	Synk-1pre	9,31	2–14	2,06	[8,94 − 9,67]	/
	Synk-1post	5,13	4–13	1,80	[4,81 − 5,45]	/
	ΔSynk-1	**4**,**17**		1,48	[3,91 − 4,43]	**< 0**,**05**
Second injection (*n* = 87)	SB-2pre	71,75	38–94	12,13		/
	SB-2post	79,35	57–98	10,56	[77,10–81,60]	/
	ΔSB-2	**11**,**75**		5,64	[20,55 − 12,96]	**< 0**,**05**
	Synk-2pre	8,56	5–13	1,87		/
	Synk-2post	4,64	0–8	1,71	[4,27 − 5,00]	/
	ΔSynk-2	**4**,**78**		1,74	[4,41 − 5,15]	**< 0**,**05**

### Iatrogenic FNP: lateral skull base subgroup population

In consideration of the entire surgical population (acoustic neuroma, parotid, cholesteatoma surgery ext.), is composed by 64 patients, those 42 patients underwent a double complete cycle of BTX-A injections. The mean pre-rehabilitation SBs of patients with iatrogenic FNP was 62.38 points, while the post-treatment value was 75.19, showing a mean growth difference of SBs after NMR/BTX-A of 12.80 (95%CI 10.81 to 14.81, *p* < 0.05). The mean value of synkinesis improvement was 4.73 (95%CI 4.19 to 5.28, *p* < 0.05): with a beginning synkinesis value (Synk-1-pre) of 9,52 and an ending synkinesis score (Synk-2-post) of 4,78. The results of the surgical cohort are shown in Table 3. The acoustic neuroma (AN) surgery group comprises 43 patients who received a single BTX-A injection and 33 cases that underwent a double cycle of injections. The AN group exhibited a mean SB of 62.79 at the outset of the rehabilitative treatment. The mean SBs following the initial injection was 71.90, with a mean increase of 9.11 points (95%CI 7.32 to 10.91, *p* < 0.005). The mean synkinesis score prior to the commencement of treatment was 9.62, while the mean synkinesis score subsequent to the conclusion of treatment was 5.25. The mean improvement was 4.37 (95%CI 3.94 to 4.8, *p* < 0.005). Furthermore, an additional improvement in SBs was observed in patients who underwent AN surgery and received a double cycle of BTX-A injections (SBs before BTX-A injection: 63.09; SBs after two BTX-A injections: 76.42). In these patients, the mean SB improvement was 13.33 (95%CI 10.95 to 15.72, *p* < 0.005). All results of patients underwent acoustic neuroma surgery and subsequent rehabilitation area showed in Table 4.

**Table 3:** Statistical analysis in surgical population and results at the end of two injections of BTX-A. ΔSB-2 = SB-2post– SB-1pre; ΔSynk-2 = Synk-2post– Synk-1pre

	Analysis	Mean		SD	CI	*P* value
Surgical population (*n* = 42)	SB-1pre	62,38		11,91	[58,66–66,00]	**/**
	SB-2post	75,19		10,12	[72,03–78,34]	**/**
	ΔSB-2	**12**,**80**		6,40	[10,81 − 14,80]	**< 0**,**05**
	Synk-1pre	9,52		2,16	[8,84 − 10,19]	**/**
	Synk-2post	4,78		1,67	[4,26 − 5,30]	**/**
	ΔSynk-2	**4**,**73**		1,76	[4,18 − 5,28]	**< 0**,**05**

**Table 4:** Statistical analysis in a subgroup surgical cohort represented by patients underwent AN surgery and its results after first and second injections of BTX-A. ΔSB-1 = SB-1post– SB-1pre; ΔSynk-1 = Synk-1post– Synk-1pre. global ΔSB = SB-2post– SB-1pre; global ΔSynk = Synk-2post– Synk-1pre

Vestibular Schwannoma First injection (*n* = 43)	SB-1pre	62,79		11,80	[59,15–66,42]	/
	SB-1post	71,90		10,83	[68,57–75,24]	/
	ΔSB-1	**9**,**11**		5,83	[7,32 − 10,91]	**< 0**,**05**
	Synk-1pre	9,62		2,12	[8,97 − 10,28]	/
	Synk-1post	5,25		1,69	[4,73 − 5,77]	/
	ΔSynk-1	**4**,**37**		1,41	[3,93 − 4,80]	**< 0**,**05**
Vestibular Schwannoma Second injection (*n* = 33)	SB-1pre	63,09		12,32	[58,72 − 67,46]	**/**
	SB-2post	76,42		10,37	[72,74–80,10]	**/**
	Global ΔSB	**13**,**33**		6,73	[10,94 − 15,72]	**< 0**,**05**
	Synk-1pre	9,87		2,08	[9,13 − 10,61]	**/**
	Synk-2post	5,00		1,52	[4,46 − 5,53]	**/**
	Global ΔSynk	**4**,**87**		1,61	[4,30 − 5,45]	**< 0**,**05**

### Latency

The onset of facial rehabilitation in the study cohort exhibited considerable heterogeneity, with a median latency of 18 months until the first BTX-A injection (ranging from 4 to 1410 months, with a mean of 119 months). A total of 73 cases started the rehabilitation protocol within a period of 24 months from the onset of the FNP. Forty-two cases began the rehabilitation protocol after a two-year interval. In fourteen patients, the data necessary for analysis were not collected. With regard to the initial injection, the former group demonstrated a SBs average improvement of 8.41 points (95%CI 7.16 to 9.66, *p* < 0.05), whereas the double-cycle injection group exhibited a mean growth of 9.38 points (95%CI 7.78 to 10.99, *p* < 0.05). Following the initial injection, synkinesis exhibited improvement in both groups, with a mean increase of 4.04 (95%CI 3.67 to 4.41) and 4.42 points (95%CI 4.05 to 4.81) respectively (*p* < 0.005). In total, data regarding the latency of the second injection were available for 79 patients, with 49 cases occurring within the first 24 months and 30 occurring after that time. The mean improvement in total SBs for both groups was 11.81 ((95%CI 10.16 to 13.48) and 11.9 (95%CI 9,67 to 14,13) (*p* < 0.05), while the reduction in synkinesis score was 4.67 (95%CI 4,14 to 5,20) and 4.93 (95%CI 4,45 to 5,51) (*p* < 0.05), respectively. A complete description of results about the latency are pointed out in Table 5.

**Table 5:** Latency of the treatment

Latency	Population	Analysis	Mean	SD	CI	*P* value
< 24 months	First injection (*n* = 73)	SB-1-pre	66,49	11,30	[63,85 − 69,13]	/
		SB-1-post	74,90	10,47	[72,45–77,34]	/
		ΔSB-1	**8**,**41**	5,35	[7,16 − 9,66]	**< 0**,**05**
		Synk-1-pre	9,47	1,99	[9,01–9,94]	/
		Synk-1-post	5,43	1,72	[5,03–5,84]	/
		ΔSynk-1	**4**,**04**	1,58	[3,67 − 4,41]	**< 0**,**05**
	Second Injection (*n* = 49)	SB-1-pre	66,73	11,02	[63,55–69,90]	/
		SB-2-post	78,55	9,11	[75,93 − 81,16]	/
		ΔSB-2	**11**,**81**	5,77	[10,15 − 13,47]	**< 0**,**05**
		Synk-1-pre	9,63	1,98	[9,06–10,02]	/
		Synk-2-post	4,95	1,55	[4,51 − 5,40]	/
		ΔSynk-2	**4**,**67**	1,84	[4,14 − 5,20]	**< 0**,**05**
> 24 months	First injection (*n* = 42)	SB-1-pre	67,83	13,94	[63,48–72,17]	/
		SB-1-post	77,21	12,73	[73,24–81,18]	/
		ΔSB-1	**9**,**38**	5,15	[7,77 − 10,98]	**< 0**,**05**
		Synk-1-pre	9,11	2,17	[8,44 − 9,79]	/
		Synk-1-post	4,69	1,88	[4,10 − 5,27]	/
		ΔSynk-1	**4**,**42**	1,21	[4,05 − 4,80]	**< 0**,**05**
	Second Injection (*n* = 30)	SB-1-pre	69,11	14,89	[63,60–74,73]	/
		SB-2-post	81,06	12,75	[76,30–85,82]	/
		ΔSB-2	**11**,**90**	5,96	[9,67 − 14,12]	**< 0**,**05**
		Synk-1-pre	8,96	1,92	[8,24 − 9,68]	/
		Synk-2-post	4,03	1,79	[3,36 − 4,70]	/
		ΔSynk-2	**4**,**93**	1,55	[4,35 − 5,51]	**< 0**,**05**

## Discussion

FNP is an insidious pathology that largely impacts the patient’s quality of life and, even when functional recovery is witnessed, sequelae can be extremely disabling. Among them the appearance of synkinesis is certainly one of the most important, with an incidence that, although it is not yet clear, stands between 9 and 55% [14–15]. Treatments for facial synkinesis include facial training mainly based on facial biofeedback retraining, chemodenervation with botulinum toxin, selective neurectomy and/or myectomy, and any combination treatment of these options [16]. It is therefore imperative that these sequelae are managed effectively. The cornerstone of therapy is the integrated treatment with chemodenervation by botulinum toxin, which is provided via NMR. The use of BTX-A for facial synkinesis was proposed after having been successfully used for the treatment of blepharospasm and facial emispasm as early as 1980 [17]. Very few works in the scientific literature provide operational guidelines on botulinum injections, especially concerning the doses (or units IU) of botulinum to be injected per single muscle group but also the injected muscles and the combination between botulinum injections and NMR [18]. The lack of standardised treatment protocols is a significant issue in this field. This study aims to provide scientific committee members with operational guidelines for the management of synkinesis in patients with peripheral FNP using botulinum toxin injections. In addition, a management rehabilitation model is proposed, emphasising defined time intervals for the resumption of logopaedic exercises from one injection to another. To the best of the authors’ knowledge, this constitutes the largest series of patients who have undergone a combined NMR/BTX-A rehabilitation programme for chronic FNP. The gender distribution of our sample is predominantly female, with an average age of 55.4 years. This highlights the substantial impact of this pathology on quality of life, particularly among socially active individuals. The predominant aetiology of FNP observed in our patient cohort is iatrogenic, aligning with the findings in the literature. Notably, the majority of cases occurred following acoustic neuroma removal. The cases included in our study are therefore extensive which is notably higher than the number of samples examined in a recent review conducted by Tavares et al. In that review, the number of patients in the studies examined ranged from 23 to 99 [19]. Due to the considerable heterogeneity of the sample population in terms of the number of injections received by each individual, our analytical approach was structured to encompass both those who had undergone a single BTX-A injection and those who had received at least two. The enhanced evaluation of SBs following both one and two injections in the general population substantiates the efficacy of this rehabilitative approach. Similarly, a statistically significant reduction was observed in the synkinesis score. The findings of our study corroborate the efficacy of botulinum toxin treatment in patients presenting with post-paralytic synkinesis, as evidenced by a review of the existing literature [20–22]. Indeed, a general enhancement in the total score as per the Sunnybrook scale has been observed, particularly in the domain of synkinesis, a finding that aligns with the results reported by Camerino et al. [23]. Furthermore, the symmetry of voluntary movement and that at rest have also improved in our cohort of patients. Other studies in the literature also aim to verify whether BTX-A treatment, by reducing the hypercontractures, would also improve synkinesis as well as its responsiveness to physical rehabilitation. Monini et al. report that the score shift in a group treated with rehabilitation alone (Group B) was similar to that obtained in the post BTX-A treatment group (Group A) in terms of improvement of facial movement. However, when considering the combined effect of BTX-A plus physical rehabilitation in this latter group, a further significant decrease of synkinesis was observed, as compared with patients who were not pharmacologically treated [24]. The increase of the score relative to the voluntary movement provides confirmation that, as a secondary effect, there is an increased activation of the musculature of the affected side when the need to control the synkinesis disappears. It is of the utmost importance that rehabilitation treatment be continued after injections to allow patients to enhance muscle excursion by capitalizing on the “window of opportunity” during which precise and isolated movements can be learned due to the absence of synkinesis [24–25].

**Fig. 1:**
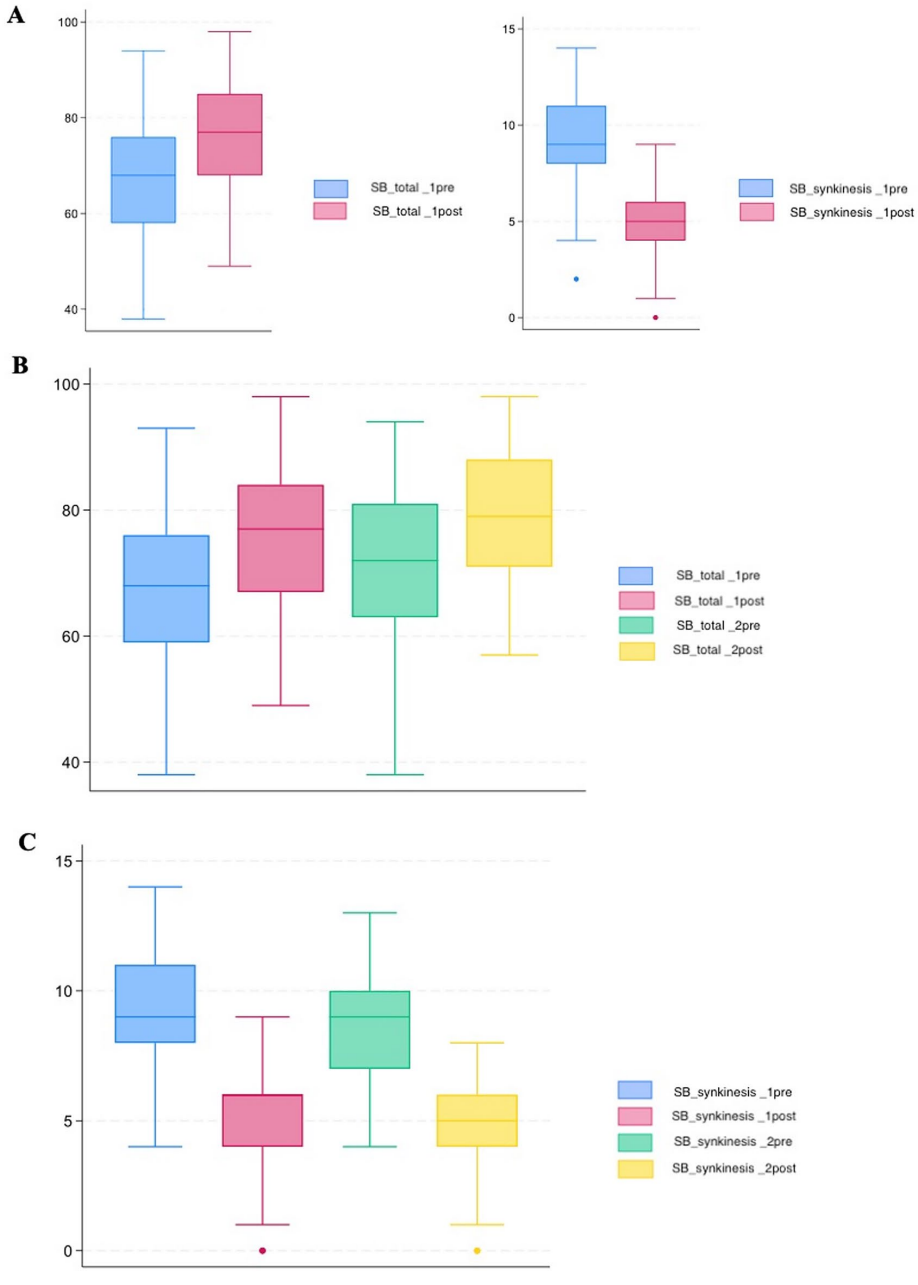
Changing in Sunnybrook and Synkinesis’ score after rehabilitation. (**A**) Considering the whole population with at least one injection (*n* = 126), figures shows an increase in SBs from the first to the second injection (box blue and red rispectively). Similarly a reduction in Synkinesis score is shown in the analyzed population. (**B**) Considering the population with two injections (*n* = 87) the SBs increases with a sinusoidal pattern. (**C**) The same for Synkinesis

In consideration of patients who have undergone two or more injections, Fig. 1 illustrates a sinusoidal evolution of the analyzed pattern. A slight decline in the parameter is observed between two cycles, following an initial increase. The explanation of this phenomenon could be the progressive decline in the efficacy of BTX-A, which coincides with the loss of the “window of opportunity” that enables the fullest possible rehabilitation. Following the administration of the second injection, a further improvement in the value was observed. It is our contention that this exponential curve represents the true strength of this kind of rehabilitation, offering the potential for significant recovery in cases of chronic FNP. Conversely, it would be unreasonable to anticipate an unending increase. It is more probable that the curve will reach a plateau that can be maintained through continued treatment, as has been observed in a few cases with six to eight injections. The efficacy of this treatment is not contingent on the underlying aetiology as both surgical and non-surgical patients demonstrate statistically significant outcomes. The results of no surgical population are showed in Table 6. The application of strict confidence intervals serves to reinforce the robustness of our findings. It is noteworthy that no discernible difference was observed based on the latency between the onset of the facial palsy and the commencement of our rehabilitation programme. This may indicate the potential for intervention in patients with a prolonged history of FNP, who are typically managed at tertiary care facilities.

**Table 6:** Statistical analysis in non-surgical population and results at the end of two injections of BTX-A. global ΔSB = SB-2post– SB-1pre; global ΔSynk = Synk-2post– Synk-1pre

	Analysis	Mean		SD	CI	*P* value
Non-surgical population (*n* = 45)	SB-1pre	72,46		11,17	[69,10–75,82]	**/**
	SB-2post	83,24		9,51	[80,38–86,10]	**/**
	Global ΔSB	**10**,**77**		4,68	[9,37 − 12,18]	**< 0**,**05**
	Synk-1pre	9,33		1,74	[8,80 − 9,85]	**/**
	Synk-2post	4,51		1,76	[3,98 − 5,04]	**/**
	Global ΔSynk	**4**,**82**		1,73	[4,30 − 5,34]	**< 0**,**05**

### Proposal treatment and injected muscles

**Fig. 2:**
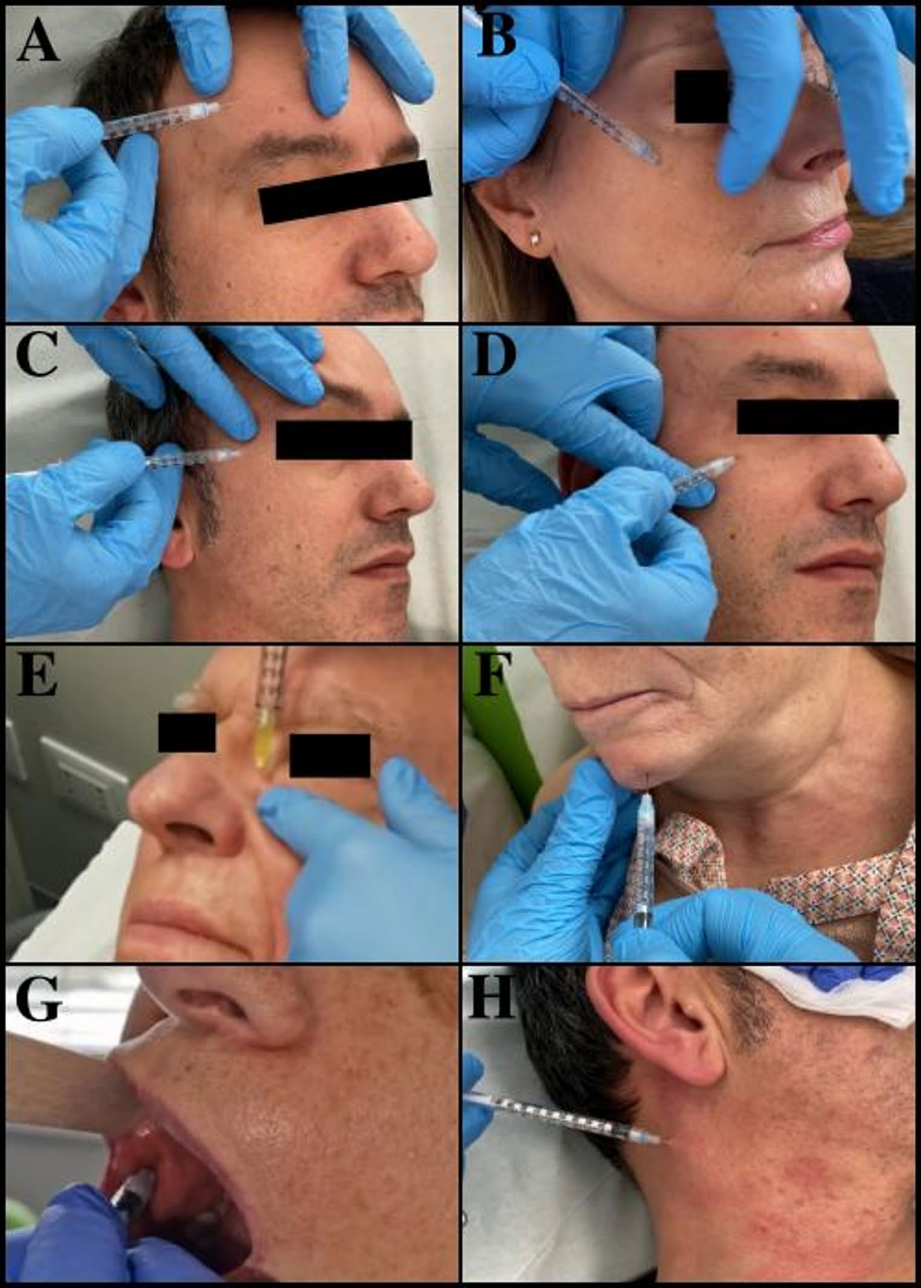
The method of BTX-A administration at the level of the frontal (**A**), zygomatic (**B**), orbicolaris oculi (**C**-**D**), levator labii superioris alaeque nasi (**E**), mentalis (**F**), orbicolari oris (**G**) and platysma (**H**) muscles is shown. It is imperative to acknowledge that not all sites of administration are encompassed in this panel, becouse of is exclusively a demostrative pourpose. Moreover, the transoral administration of the orbicolaris oris (**G**) constitutes a preliminary experience in our centre as delineated in the ensuing discourse, and is not included in the statistical outcomes of this study

**Fig. 3:**
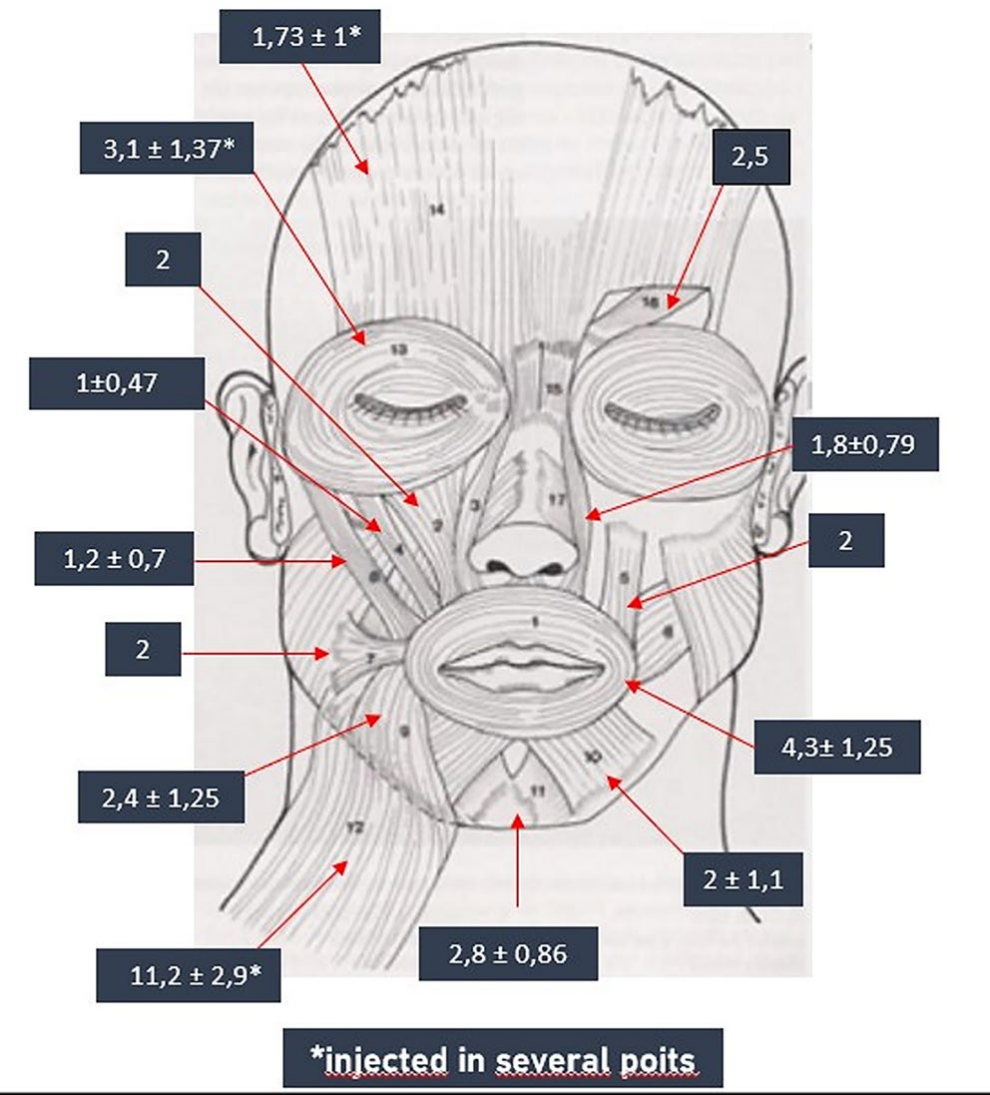
The mean unit administered at the first BTX-A injection per targeted muscle on the affected side is showed

**Fig. 4:**
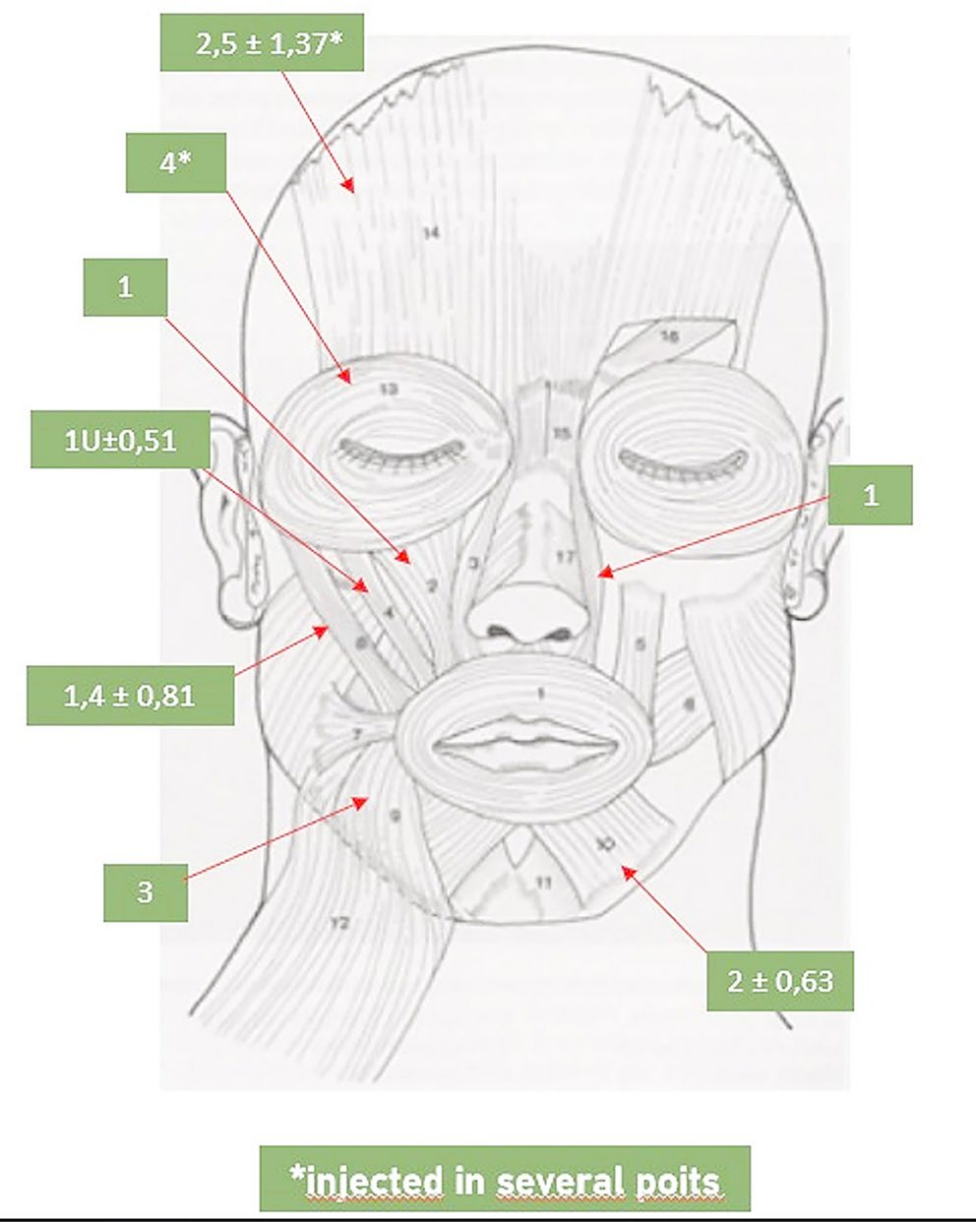
The mean unit administered at the first BTX-A injection per targeted muscle on the healty side is showed

The treatment has been proposed for all patients with synkinesis, regardless of the specific entity involved. Some authors have proposed the implementation of treatment exclusively in instances of moderate to severe synkinesis [19, 31]. It is reasonable to treat the synkinesis once 12 months have elapsed since the acute event, even if mild, to avoid interfering with reinnervation. Furthermore, a time-modulated treatment with lower doses and fewer muscles injected should be considered. A review of the literature revealed that the most commonly injected muscles are the frontalis, corrugator supercilia, orbicularis oculi, levator labii superioris, levator labii superioris alaeque nasi, major zygomaticus, orbicularis oris, risorius, depressor anguli oris, depressor labii inferioris, mentalis and platysma [19]. Our experience has shown that the most targeted muscles on the affected side are the platysma, orbicularis oculi, mentalis, levator labii superioris, levator labii superioris alaeque nasi and depressor anguli oris. Conversely, on the healthy side, the frontalis, depressor labii inferioris and depressor anguli oris muscles are more frequently injected in order to achieve facial symmetry at rest. As illustrated in Fig. 2, the mode and directionality of administration of some of the injected subsites are exhibited. A detailed account of the mean unit administered at the first BTX-A injection per targeted muscle on the affected and healthy sides is illustrated in Figs. 3 and 4, respectively. Injecting the remaining muscles is an uncommon practice, as it is thought that this does not provide a significant additional benefit. Furthermore, there is an increased risk of treatment complications for the patient. To illustrate, the muscles responsible for a more pronounced nasolabial groove may include the major zygomatic, the risorius, the levator labii superioris, and the levator labii superioris alaeque nasi. In the case of this particular issue, the treatment is typically limited to the latter muscle, as the treatment of the zygomatic complex does not appear to yield superior outcomes, with the potential for complications such as a reduction in the range of the smile and excessive collapse of the zygomatic region. Indeed, some authors have even advised against such treatment [32]. Similarly, the treatment of the levator labii superioris carries a significant risk of the upper lip falling. The favourable outcomes observed, along with the low incidence of complications, appear to lend support to our clinical practice. In the course of our case studies, no patients underwent injection of BTX-A into the nasalis, procerus, and buccinator muscles. In a one-year prospective cohort study of 84 patients, Patel et al. demonstrated that buccinator treatment did not result in a change in Synkinesis Assessment Questionnaire (SAQ) scores relative to no-buccinator treatment cycles. Nevertheless, a more detailed examination of questions pertaining to buccinator function (specifically, facial tightness and lip movement) revealed a notable enhancement in these areas with the incorporation of buccinator treatment [33]. Similarly, Wei et al. demonstrated comparable outcomes in a cohort of 42 patients, with a notable improvement in SAQ scores from a mean of 66.6 pre-injection to 45.0 post-injection. The mean total dose of botulinum administered to the buccinator per session was 2.0 units (range, 0.6–2.5 units) [34]. This raises the interesting question of how this prominent midfacial muscle should be treated. In patients presenting with facial synkinesis, the administration of botulinum toxin injections to the buccinator muscle has been demonstrated to result in a notable improvement in the subjective assessment of disease severity. This intervention can be safely employed in patients who have been selected according to the appropriate criteria and who are undergoing injections of other synkinetic facial musculature in synergy with facial rehabilitation. Patients who experience a persistent sensation of tightness at the oral commissure and who present with distortion or retraction of the oral commissure, difficulty controlling a food bolus with chewing or frequent biting of the cheek during chewing, may be considered optimal candidates for botulinum toxin injection into this muscle. Our team does not generally perform buccinator muscle injections due to the potential for pain and complications arising from deeper administration. Instead, BTX-A administration is performed trans-orally at the level of the orbicularis oris muscle of the mouth in these patients. This manoeuvre is preceded by an objective assessment of the hypercontracted state of the muscle by means of bimanual palpation of the cheek, and provides a favourable subjective response from patients.

A review of our data reveals a consistent pattern in the frequency of injections across the various muscles and sessions. This is particularly predictable for the platysma and mentalis muscles, where rehabilitation strategies are limited by the absence of specific exercises. In contrast, the orbicular muscle of the eye suffers from involuntary blinking, which can partially affect the control of synkinesis. Furthermore, there has been a gradual increase in the treatment of contralateral muscles on the healthy side as the follow-up period has progressed. This outcome may reflect the fact that controlateral musculature treatment, particularly for aesthetic purposes, is initiated when paralysis is more stable and the effects of BTX-A on the paretic side have been tested safely. The frequency of treatment for the depressor complex of the lower lip has been observed to decrease in favour of treatment of the depressor anguli oris on the healthy side. This approach has been found to offer similar benefits in terms of facial symmetry with a reduced risk of lower lip ptosis. The frontalis muscle is another frequently targeted muscle on the healthy side. In our practice, we have noted a slight increase in the number of injections being performed. In addition, contralateral muscles may be injected, as documented by Shinn et al., who treated the corrugator supercilii, zygomatic, and mentalis muscles [27]. However, this approach is not commonly employed in our clinical practice.

### BTX-A dosage and time range

As for the injection techniques used, most are in line with those described in the literature. However, for the treatment of the orbicularis oculi muscle, several options are presented to prevent the occurrence of complications such as eyelid ptosis, diplopia and lagophthalmos. It is generally agreed the contraindication in treating the medial portion of that muscle due to the risk of toxin diffusion at the level of inferior rectus and inferior oblique muscles and consequent diplopia onset. More controversial is the injection of the superolateral portion of orbicularis oculi muscle, in proximity to the levator palpebrae superioris muscle, potential cause of ptosis. From Della Toffola et al. they treat this muscle through a single inferolateral injection and Tavares et al. recommend using low dosages when injecting it above [19, 20]. Our technique, associated with good results and absence of eye complications, provides for an injection of the orbicularis oculi in two points, superolaterally and inferolaterally with an increasing dose at a higher level to avoid lagophthalmos. In our opinion it is therefore effective and safe as long as the upper infiltration is conducted keeping very lateral and therefore far from the levator palpebrae superioris muscle. The average dose used in the single session was 15,7 IU per patient, as well as the dose per injection site ranged between 1 and 5 IU, with the exception of orbicularis oculi (up to 12 IU) and platysma muscles (up to 16 IU). The analysis of the population reveals a notable progressive increase of the dosage of BTX-A over time, a trend that has been previously documented by other researchers [27]. This increase appears to be associated with the rise in the number of treated muscles as outlined by Alipour et al., rather than with the increase in units of botulinum toxin injected into specific muscle groups, particularly the platysma and the orbicularis oculi, with a lesser degree of influence observed in the levator labii superioris alaeque nasi [28]. As indicated by Shinn et al., the mean dose demonstrates a gradual elevation in the muscles of the upper and middle regions of the face and neck, whereas it remains unaltered in those of the lower face [27]. It is standard practice to commence treatment with a minimal dosage, with the objective of gauging the patient’s tolerance. Thereafter, the dosage is incrementally augmented in the absence of adverse effects and in accordance with the clinical response. The observation of only minor and transient side effects during the initial session substantiates the efficacy of this approach.

Despite some authors reporting the necessity for a reduced dosage of botulinum toxin in patients with idiopathic paralysis in comparison to those with other aetiologies, no statistically significant difference in dosage was observed when BTX-A was used in our cohort [28].

The mean time interval between sessions was 6 months. The duration of the effect of BTX-A in the treatment of facial synkinesis thus appears to be longer than that of the same toxin used for aesthetic purposes, which has been observed to persist for a period of 4 to 6 months, in accordance with the studies conducted by Azuma and do Nascimento Remigio [29–30]. Other studies have reported a mean duration of Onabotulinumtoxin A effect that ranges from 66 days to four months [19]. In the remaining literature, a shorter average interval (2–4 months) is reported between one session and another. However, the heterogeneity of the rehabilitation treatments undertaken precludes an adequate comparison of the data. It is likely that the reduction of this interval is due to the tendency to anticipate treatments in the last year, in order to intervene when the synkinesis are still slight and thus allowing the injection of fewer units and fewer muscles.

### Final tips

- **Muscles and Botulinum Toxin Dosages**: The platysma and orbicularis oculi require higher doses of botulinum toxin, and multiple sessions may be needed to determine the optimal dosage. Caution is essential when increasing the dose due to resistance synkinesis in the eye area and at the level of levator labii superioris alaeque nasi muscle. Overdosing can weaken the muscles and lead to the recurrence of eyelid dysfunction.
- **Synkinesis and Follow-up**: Even after clinical recovery, synkinesis may develop. A 1-year follow-up is recommended to monitor progress and design an appropriate rehabilitative protocol for late-onset synkinesis.

### Limits

Being a retrospective observational study, the results of our research may suffer from some bias. Compared to randomized controlled trials, observational studies have, by construction, problems of internal validity and a possible bias resulting from patient selection.

Although patients express satisfaction with and continue to receive treatment with botulinum toxin, our data are not supported by the compilation of a subjective questionnaire such as the SAQ, which is considered a reliable tool and useful in evaluating important sequelae, such as synkinesis. It is also true that in Italy, the aforementioned questionnaire was only recently validated by the scientific community, which justifies its use among patients of our facial nerve office only in recent times.

## Conclusions

Botulinum toxin type A (BTX-A) has proven effective in reducing synkinesis and improving facial symmetry, especially when applied to both the affected and contralateral healthy side. It also supports the recovery of voluntary movement, provided the patient undergoes consistent and appropriate rehabilitation. Neuromuscular retraining (NMRT) enhances functional recovery by encouraging symmetrical, repeated, and tailored movements for specific muscle groups. Combining NMRT with BTX-A injections in synkinetic regions shows promising results. An integrated, long-term treatment strategy is essential, starting with lower doses and gradually increasing to the optimal dosage, allowing for safe treatment of areas requiring higher doses, such as the orbicularis oculi and platysma. In the absence of standardized protocols, this study offers a comprehensive guide for managing BTX-A treatment in patients with synkinesis following facial nerve paralysis (FNP).
