# Correction: Rehabilitation of facial nerve palsy combining neuromuscular retraining and botulinum toxin A injection: a tertiary referral centre experience and a new working protocol proposal

**DOI:** 10.1007/s00405-025-09722-0

**Published:** 2025-10-14

**Authors:** Marco Bonali, Federico Calvaruso, Andrea Tozzi, Cinzia Del Giovane, Federica Nizzoli, Elena Reggiani, Alfredo Lo Manto, Martina Silvestri, Daniele Marchioni, Giuseppe Ferrulli, Claudio Melchiorri

**Affiliations:** 1 Department of Otorhinolaryngology - Head and Neck Surgery, University Hospital of Modena, 41124 Modena, Italy; 2 Department of Otorhinolaryngology - Head and Neck Surgery, Ospedale Infermi, 47900 Rimini, Italy; 3 Department of Medical and Surgical Sciences for Children and Adults, University Hospital of Modena, 41124 Modena, Italy; 4 Institute of Primary Health Care (BIHAM), University of Bern, 3012 Bern, Switzerland; 5 Otolaryngology Unit, Department of Surgery, Azienda USL-IRCCS di Reggio Emilia, 42123 Reggio Emilia, Italy

**Correction: European Archives of Oto-Rhino-Laryngology (2025) 282:3757–3769**

10.1007/s00405-025-09465-y.

In the original version of this article, the given and family names of Marco Bonali, Federico Calvaruso, Cinzia Del Giovane, Federica Nizzoli, Elena Reggiani, Alfredo Lo Manto, Martina Silvestri, Daniele Marchioni, Giuseppe Ferrulli and Claudio Melchiorri were incorrectly structured. The names were displayed correctly in all versions at the time of publication.

The original article has been corrected.

